# Insights into the link between drug use and criminality: Lifetime offending of criminally-active opiate users

**DOI:** 10.1016/j.drugalcdep.2017.07.024

**Published:** 2017-10-01

**Authors:** Matthias Pierce, Karen Hayhurst, Sheila M. Bird, Matthew Hickman, Toby Seddon, Graham Dunn, Tim Millar

**Affiliations:** a Centre for Mental Health and Safety, University of Manchester, 4th Floor, Ellen Wilkinson Building, Oxford Road, M13 9PL, UK; b MRC Biostatistics Unit, Institute of Public Health, University Forvie Site, Robinson Way, Cambridge, CB2 0SR, UK; c School of Social and Community Medicine, University of Bristol, Canynge Hall, 39 Whatley Road, Bristol, BS8 2PS, UK; d School of Law, University of Manchester, 4.46A Williamson Building, Oxford Road, M13 9PL, UK; e Centre for Biostatistics, University of Manchester, Jean McFarlane Building (First Floor), Oxford Road, M13 9PL, UK

**Keywords:** Offending, Opiate use, Life-course offending

## Abstract

**Background:**

We test whether the offending trajectory of those who test positive for opiates is greater than test-negative controls and whether the relationship is constant both prior to, and post, opiate initiation. We consider whether these relationships differ according to gender and offence type.

**Methods:**

The study provides an analysis of historical offending records in adults linked to test results for opiate and cocaine metabolites. Those testing positive for opiates were linked to treatment records to retrieve data on age of opiate initiation. Rate ratios (RR) were calculated to compare opiate positive testers to opiate and cocaine negative controls, separately by gender and adjusting for age and birth cohort. Age of opiate initiation was included in a second model as a time-dependent variable. Within-subject clustering was accounted for using generalised estimating equations.

**Results:**

Opiate-positive cases had higher rates of offending than test-negative controls, both prior to, and post, opiate initiation. Initiation of opiate use increased the RR by 16% for males but doubled it for females. The RR increase in non-serious acquisitive crime was greater than that seen in serious crime. For males only, opiate initiation narrowed the difference in violent offending rate between cases and controls. A larger offending increase was associated with opiate initiation in female, compared to male, users.

**Conclusions:**

For most crime categories, the difference between groups is exacerbated by opiate initiation. The findings indicate that opiate prevention initiatives might be effective in reducing offending, particularly among females.

## Highlights

• Over the life-course, opiate users have elevated rates of acquisitive offending.
• This association exists prior to opiate-initiation.
• Opiate initiation escalates the difference between opiate users and non-users.
• This escalation is greater for females and for non-serious acquisitive offences.

## 1 Introduction

Those dependent on heroin, and other opiates, are disproportionately involved in criminal activity (Bennett et al., 2008); in particular, acquisitive offending (crimes committed for financial gain) (Bukten et al., 2011, Pierce et al., 2015). The drugs-crime association is an important driver of UK policy, reflected in its prominence in the drug strategies of successive governments (HM Government, 2008, Home Office, 2010). Explanations of this association fall into three groups:

1. Forward causation – drug use causes crime either through the need to: (a) fund drug use through economic necessity (Bennett et al., 2008); or (b) because of psychopharmacological changes precipitated by drug ingestion (Boyum and Kleiman, 2002, Brownstein, 2016, White and Gorman, 2000).
2. Reverse causation – involvement with crime leads to drug use: opportunities for drug use increase with involvement in criminal behaviour (Hammersley et al., 1989).
3. Confounding – crime and drug use share a common (set of) cause(s): there is no direct causal relationship; rather drug use and crime co-occur because of a common cause or set of causes (Seddon, 2006, Seddon, 2000).

The underlying causal mechanism(s) is likely to be more complex than these explanations suggest (Bennett and Holloway, 2009, Seddon, 2000). Our previous work has highlighted the need for longitudinal studies with a non-drug user comparison group to examine the natural history of drug use and offending (Hayhurst et al., 2017). Whilst cross-sectional studies can provide information on the extent of the drug-crime association and its strength for different subgroups and offences, the aetiological debate requires longitudinal data to establish the timing of events and to gain knowledge on how the differences between users and non-users evolves over a person’s lifetime.

Current evidence about the development of drug use and offending is constrained by design flaws in published studies, particularly the absence of suitable control groups. Our recent review of the evidence base on pathways through opiate use and offending (Hayhurst et al., 2017) highlighted that research has focused on comparing offending that occurs prior to the initiation of drug use with offending that occurs thereafter. A typical example is the study by Anglin and Speckart (1988), which examined the criminal records and clinical data of male methadone patients. Most studies which make this comparison find that offending rates are substantially higher after drug-use initiation (Hayhurst et al., 2017). This pre/post design fails to separate the effects of initiation from the effects of other factors which might also be related to offending, in particular, age, which correlates strongly with offending. In general population samples, offending rates tend to peak during late adolescence (Sweeten et al., 2013) which coincides with the age of drug-use initiation. For example, a large proportion (45%) of users in treatment services in the North West of England report age at first use of heroin between 15 and 19 years of age (Advisory Council on the Misuse of Drugs, 2006). To disentangle the age effects from those of drug-use initiation, it is crucial to control for age, using an appropriate control group. Similarly, gender is known to be a strong influence on offending trajectories and whilst some studies have shown the pre/post contrast is greater for females (Degenhardt et al., 2013), the lack of adequate comparator groups limits the inferences which can be drawn.

This paper reports a retrospective cohort analysis to compare the historical offending trajectory of offenders according to drug test result. Prior analysis on this cohort considered offending rates in the two years prior to drug-test and found that testing positive for opiates was a greater predictor of excess offending than testing positive for cocaine. We therefore focus on opiate use, by comparing the historical offending trajectory of offenders who test positive for opiate use (opiate positives) with a control group who test negative for both opiate and cocaine use (test-negatives). This comparison is performed for all offences committed and for three offence categories (serious acquisitive, non-serious acquisitive, violent) whilst controlling for age and birth cohort, and separately by gender. Information about the age of first opiate use is used to consider whether the contrast between opiate positives and test-negatives is similar both before, and after, the initiation of opiate use. The following hypotheses are considered:

1. Opiate positives exhibit higher rates of offending than negative testers prior to opiate positives’ initiation of opiate use;
2. The initiation of opiate use exacerbates the level of offending compared to negative testers;
3. The effect of opiate-use initiation is different for males and females.
4. The effect of opiate-use initiation differs by crime type.

## 2 Methods

### 2.1 Data

The analysis cohort was identified from those who received a saliva drug test for opiate and cocaine metabolites following arrest, as recorded by the Drug Test Record (DTR), over the period 1st April 2005 to 31st March 2009. Age at drug-use initiation was obtained for the subset also recorded in the English National Drug Treatment Monitoring System (NDTMS) over the same period. Cohort members’ complete recorded offending history (up to 31st March 2009) was extracted from the Police National Computer (PNC).

The cohort was defined from each subject’s first drug-test record which satisfied the following criteria: (1) the subject was 18–39 years old; (2) the test was completed and undisputed; and (3) the subject was charged and sanctioned following their arrest, as evidenced from a contemporaneous PNC record. This cohort has been described in detail elsewhere (Pierce et al., 2015), with the modification here of a lower upper age range and the exclusion of Wales. The age range restriction was applied since the profile of individuals whose offending persists into their 40s may be atypical (Moffitt, 1993, Moffitt and Caspi, 2016). Those drug-tested in Wales were excluded because NDTMS has coverage for England only. From the analysis cohort, we define opiate-positive *cases* as those who, on arrest, tested positive for opiates and negative tester *controls* as those who tested negative for opiates and cocaine.

The DTR records a mandatory saliva test for opiate and cocaine (crack or powder form) metabolites following arrest for a ‘trigger’ offence (pre-defined as associated with problem drug use), or at the discretion of the police officer in charge of the custody area. Trigger offences are: theft; robbery; burglary; vehicle theft; supply or possession of cocaine or heroin (Home Office, 2011). Data are retained on positive and negative saliva test results, test dates, reason for test and basic demographic information. Those who test positive are required to attend an initial assessment with a drugs worker who will help the user seek treatment and other support.

The PNC is an operational database recording all UK arrests that result in a criminal charge. We consider the subset which resulted in a conviction or a caution, reprimand or warning (i.e., *sanctioned* offences). All sanctioned offences committed by the individual were included, from age 10 (the age of criminal liability in England) up to the two weeks prior to the drug test. We excluded this two-week period to negate the effect of the specific offence which resulted in the drug test.

NDTMS records information about individuals who seek treatment for psychoactive substance-related problems by National Health Service and third-sector providers (Marsden et al., 2009). It includes information about the age at which patients first used the drug they sought treatment for. We linked cases in the analysis cohort to NDTMS records for subjects treated for opioid dependence between 1st April 2005 and 31st March 2009. NDTMS has national coverage, so every subject who received drug treatment in this period should have a record. The analysis was conducted on a complete case basis and those with missing age-of-initiation were described (see Appendix A in the Supplementary material).

Linkage between datasets was based on a *minimal identifier* (initials, date of birth and gender). Additionally, the PNC includes a unique identifier (PNC-ID). Those minimal identifiers with multiple PNC-IDs were excluded from the analysis, as this was taken as indicating a duplicated record. All identifiers were anonymised prior to their release to the study team to ensure that features of the original data could not be discerned.

### 2.2 Statistical analysis

In order to compare life-course offending between opiate-positive cases and negative test controls, offence counts per individual were grouped into 1-year age bands and a generalised estimating equation (GEE) was fitted to the data. GEEs account for correlations within clustered observations; in this analysis, offence counts belonging to the same individual. We used a log-link function and included ‘time-at-risk’ as an offset, so that the model parameters are interpreted as population-averaged estimates of the log increase in offending rate associated with a unit change in the variable. The exponential of this term is interpretable as a rate ratio (RR). The model employed an exchangeable correlation structure.

The analysis considered two models. Using the whole cohort, the first model estimated the RR associated with being an opiate user, whilst controlling for age (in years: linear and quadratic terms) and birth cohort (year of birth categorised into: <1975, 1975–1979, 1980–1984, 1985+).

The second model included only those cases that had an NDTMS record. This analysis included the same variables present in the first model with the addition of the time-dependent variable ‘initiated opiate use’, which changed value from zero to one for the year where the user declared initiating opiate use, as per their NDTMS record. Within this model there are two parameters of interest: (1) being an opiate-positive case; and (2) the initiation of opiate use. In a model with both present, the first is interpreted as the RR of the change in offending, associated with being opiate positive, prior to opiate initiation; the second as the change in the RR associated with opiate initiation. Linear combinations of these parameters can be used to derive the estimated change in offending rate associated with opiate-user status, post-initiation of drug use. For example, if the RR associated with being a case is 1.5 and the effect of ‘initiation of opiate use’ is 2 then the RR comparing cases and controls prior to initiation is 1.5 and the RR post-onset of opiate use is 3.0. For ease of interpretation we include all three estimates.

The analysis considered the categories of violent and acquisitive offences, with the latter disaggregated further into ‘serious’ and ‘non-serious’ acquisitive offences according to definitions used in local government reporting (Audit commission, 2010). Sub-categories which fall under serious acquisitive crimes are: burglary; robbery; theft of a vehicle; and theft from a vehicle. Those that fall under non-serious acquisitive crimes are: prostitution; theft from a person; theft from a shop; other theft; fraud and forgery; and drug supply offences. The offences that comprise these sub-categories are detailed in Appendix B (Supplementary material).

A number of those who tested positive for opiates also tested positive for cocaine. Our prior analysis (Pierce et al., 2015) demonstrated that those who tested positive for both drugs had rates of offending higher than those who tested positive for opiates only. As a sensitivity analysis, we therefore consider whether the effect of opiate-use initiation was similar in those who tested positive for opiates only and those who tested positive for both drugs (see Appendix C in the Supplementary material).

## 3 Results

### 3.1 Cohort description (Table 1)

The analysis cohort consisted of 18,965 opiate-positive cases and 78,838 test-negative controls. A quarter of both groups were female. Cases were older at their drug test (p < 0.001) and younger at their first recorded offence (p < 0.001). Cases were more likely to have a conviction for a serious acquisitive offence at this date (p < 0.001) and less likely to have a conviction for a violent offence (p < 0.001).

**Table 1:** Description of cohort by DTR test.

	*Opiate positive (N* *=* *18,965)*	*Test negative (N* *=* *78,838)*	p-value*
Index test result (%)			
Negative opiates and cocaine	(0)	78,838 (100)	
Opiate positive, cocaine negative	7259 (38)	(0)	
Opiate positive, cocaine positive	11,706 (62)	(0)	

Gender (%)			0.23
Female	4614 (24)	18,854 (24)	
Male	14,351 (76)	59,984 (76)	

Median age at test [IQR]	29.9 [25.8–34.4]	24.0 [20.2–30.0]	<0.001

Median number of past crimes [IQR]	25 [8–50]	3 [0–13]	<0.001

Median age at first recorded offence [IQR]	16.9 [14.7–19.5]	17.1 [14.7–20.3]	<0.001

Type of crime at first recorded offence (%)*			
Violence offences	1300 (10)	6713 (14)	<0.001
Serious acquisitive	2595 (21)	7693 (16)	<0.001
Non-serious acquisitive	5059 (40)	17,390 (37)	<0.001
Other	3593 (29)	15,226 (32)	<0.001

Missing age of initiation (%)	6238 (33)		
No linked NDTMS record	4530 (24)		
Missing age of initiation within NDTMS record	1708 (9)		

Median age of initiation [IQR]	19 [17,23]		
Males	19 [17,23]		
Females	19 [16,22]		

^**^Categories not mutually exclusive.

* p-value from chi-squared test, for test of proportions, or the Mann-Whitney test, for test of medians.

Sixty-seven per cent of opiate-positive cases had complete data on age-of-initiation. The majority of missing data were due to cases not having a linked treatment record (see Appendix A in the Supplementary material). The median age of initiation was similar for men and women.

### 3.2 Offending history (Table 2)

**Fig. 1:**
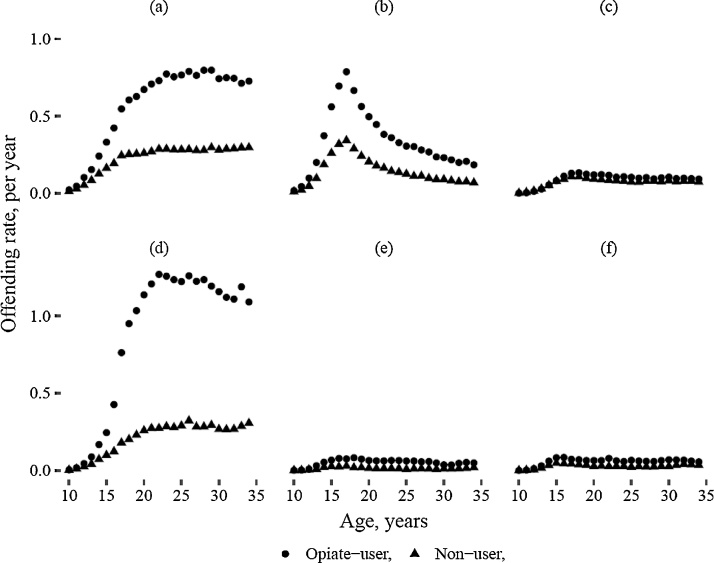
Offending rates, per year by age, opiate users and non-users for: (a) male, non-serious acquisitive offences; (b) male, serious acquisitive offences; (c) male, violent offences; (d) female, non-serious acquisitive offences; (e) female, serious acquisitive offences; (f) female, violent offences.

In total, the cohort had 1.6 million sanctioned offences. For men, the rate of historical offending for opiate-positive cases was almost double that for test-negative controls (rate per year, opiate users: 1.82; non-users: 0.91; p < 0.001); the rate for opiate-positive females was more than four times that for test-negative females (opiate users: 1.38; non-users: 0.33; p < 0.001). For both male and female opiate users, the rate of offending was lower prior to initiation of opiate use compared to post-initiation. For males and females, the rate of violent and serious acquisitive offending peaked during the late teens, whilst the rate of non-serious acquisitive offences had a later peak (Fig. 1a and b).

**Table 2:** Offending rates for four categories of offences.

			All crimes		Non-serious acquisitive crimes		Serious acquisitive crimes		Violent crimes	
Gender	Category	person years follow-up	Number	Rate [95% CI]	Number	Rate [95% CI]	Number	Rate [95% CI]	Number	Rate [95% CI]
Male	non-users	923,663	837,019	0.91 [0.90, 0.91]	176,783	0.19 [0.19, 0.19]	150,177	0.16 [0.16, 0.16]	61,730	0.07 [0.07, 0.07]
	Opiate users	290,007	528,153	1.82 [1.82, 1.83]	153,031	0.53 [0.53, 0.53]	103,654	0.36 [0.36, 0.36]	25,247	0.09 [0.09, 0.09]
	Pre-initiation	96,491	115,682	1.20 [1.19, 1.21]	25,285	0.26 [0.26, 0.27]	34,317	0.36 [0.35, 0.36]	6672	0.07 [0.07, 0.07]
	Post-initiation	97,788	270,885	2.77 [2.76, 2.78]	91,148	0.93 [0.93, 0.94]	40,917	0.42 [0.41, 0.42]	10,796	0.11 [0.11, 0.11]
	Initiation missing	95,728	141,586	1.48 [1.47, 1.49]	36,598	0.38 [0.38, 0.39]	28,420	0.30 [0.29, 0.30]	7779	0.08 [0.08, 0.08]

Female	non-users	304,612	100,525	0.33 [0.33, 0.33]	51,518	0.17 [0.17, 0.17]	4194	0.01 [0.01, 0.01]	8192	0.03 [0.03, 0.03]
	Opiate users	87,373	120,336	1.38 [1.37, 1.39]	66,637	0.76 [0.76, 0.77]	4509	0.05 [0.05, 0.05]	4840	0.06 [0.05, 0.06]
	Pre-initiation	32,839	15,139	0.46 [0.45, 0.47]	8335	0.25 [0.25, 0.26]	1096	0.03 [0.03, 0.04]	1149	0.03 [0.03, 0.04]
	Post-initiation	29,807	80,056	2.69 [2.67, 2.70]	44,767	1.50 [1.49, 1.52]	2451	0.08 [0.08, 0.09]	2523	0.08 [0.08, 0.09]
	Initiation missing	24,727	25,141	1.02 [1.00, 1.03]	13,535	0.55 [0.54, 0.56]	962	0.04 [0.04, 0.04]	1168	0.05 [0.04, 0.05]

### 3.3 Comparison of offending trajectory opiate-user cases vs. non-user controls (Table 3)

#### 3.3.1 Model 1: change in offending trajectory

Controlling for age, age-squared and age-cohort, male opiate positive’s prior total offending rate was double that for test-negatives (Rate Ratio: 1.99, 95% CI: 1.96–2.01); for females, it was over four times greater (RR: 4.59, 95% CI: 4.48–4.69). There was a relative increase in all categories of offending associated with being opiate-positive, with a greater increase for females than for males. The greatest increase associated with being an opiate–positive was for females and for the category non-serious acquisitive offending (RR: 4.79, 95% CI: 4.66–4.91). The lowest increase was for males and for the violent offences category.

**Table 3:** Results of Generalised Estimating Equation analysis comparing historical offending rates of opiate users and non-users using whole sample (Model 1, N = 97,803) and those with complete data on age of initiation of opiate use (Model 2, N = 91,565), separately for males and females and for four categories of offences.

		Male				Female			
		Model 1^b^		Model 2^c^		Model 1^b^		Model 2^c^	
Offence category	Variable	RR	95% CI	RR	95% CI	RR	95% CI	RR	95% CI
All crimes	Opiate users vs. non-users	1.99	[1.96, 2.01]	–	–	4.59	[4.48, 4.69]	–	–
	Initiation of opiate use	–	–	1.16	[1.15, 1.17]	–	–	2.00	[1.95, 2.05]
	Users (pre-onset) vs. non-users	–	–	2.00	[1.97, 2.03]	–	–	2.80	[2.71, 2.90]
	Users (post-onset) vs. non-users	–	–	2.32	[2.29, 2.35]	–	–	5.61	[5.47, 5.75]
	Age^a^	1.92	[1.92, 1.93]	1.90	[1.90, 1.91]	2.53	[2.51, 2.55]	2.32	[2.30, 2.34]
	Age-squared^a^	0.77	[0.77, 0.78]	0.77	[0.77, 0.77]	0.78	[0.78, 0.78]	0.79	[0.79, 0.79]
	Age-cohort								
	<1975	0.75	[0.74, 0.76]	0.74	[0.73, 0.75]	0.62	[0.60, 0.64]	0.68	[0.66, 0.70]
	1975–1979	0.86	[0.85, 0.87]	0.85	[0.84, 0.86]	0.78	[0.76, 0.80]	0.82	[0.79, 0.84]
	1980–1984	Ref		Ref		Ref		Ref	
	1985+	1.32	[1.30, 1.34]	1.33	[1.31, 1.35]	1.76	[1.71, 1.82]	1.71	[1.65, 1.76]

Non-serious acquisitive	Opiate users vs. non-users	2.65	[2.61, 2.69]	–	–	4.79	[4.66, 4.91]	–	–
	Initiation of opiate use	–	–	1.72	[1.69, 1.75]	–	–	2.18	[2.11, 2.25]
	Users (pre-onset) vs. non-users	–	–	1.97	[1.92, 2.02]	–	–	2.73	[2.62, 2.85]
	Users (post-onset) vs. non-users	–	–	3.39	[3.34, 3.45]	–	–	5.95	[5.78, 6.12]
	Age	1.85	[1.84, 1.85]	1.74	[1.73, 1.75]	2.46	[2.43, 2.48]	2.23	[2.20, 2.25]
	Age-squared	0.83	[0.83, 0.83]	0.83	[0.83, 0.83]	0.76	[0.76, 0.77]	0.78	[0.77, 0.78]
	Age-cohort								
	<1975	0.87	[0.85, 0.89]	0.92	[0.90, 0.93]	0.80	[0.78, 0.83]	0.90	[0.87, 0.93]
	1975–1979	0.95	[0.93, 0.97]	0.96	[0.94, 0.98]	0.88	[0.85, 0.91]	0.93	[0.89, 0.96]
	1980–1984	Ref		Ref		Ref		Ref	
	1985+	1.08	[1.05, 1.10]	1.05	[1.02, 1.07]	1.30	[1.25, 1.35]	1.26	[1.21, 1.32]

Serious acquisitive	Opiate users vs. non-users	1.84	[1.81, 1.87]	–	–	4.11	[3.85, 4.38]	–	–
	Initiation of opiate use	–	–	1.25	[1.22, 1.27]	–	–	1.76	[1.62, 1.92]
	Users (pre-onset) vs. non-users	–	–	1.87	[1.82, 1.91]	–	–	3.16	[2.88, 3.46]
	Users (post-onset) vs. non-users	–	–	2.33	[2.27, 2.38]	–	–	5.58	[5.19, 6.00]
	Age^a^	1.16	[1.15, 1.16]	1.11	[1.11, 1.12]	1.39	[1.36, 1.42]	1.27	[1.23, 1.30]
	Age-squared^a^	0.66	[0.66, 0.66]	0.65	[0.64, 0.65]	0.81	[0.80, 0.82]	0.81	[0.80, 0.83]
	Age-cohort								
	<1975	0.83	[0.81, 0.84]	0.73	[0.71, 0.75]	0.75	[0.69, 0.82]	0.84	[0.77, 0.93]
	1975–1979	1.40	[1.37, 1.43]	1.39	[1.36, 1.42]	0.83	[0.76, 0.91]	0.90	[0.82, 0.99]
	1980–1984	Ref		Ref		Ref		Ref	
	1985+	1.05	[1.02, 1.07]	1.06	[1.04, 1.09]	1.44	[1.31, 1.57]	1.46	[1.33, 1.61]

Violent offences	Opiate users vs. non-users	1.39	[1.36, 1.42]	–	–	2.42	[2.30, 2.55]	–	–
	Initiation of opiate use	–	–	0.75	[0.72, 0.77]	–	–	1.04	[0.96, 1.13]
	Users (pre-onset) vs. non-users	–	–	1.79	[1.72, 1.85]	–	–	2.51	[2.31, 2.72]
	Users (post-onset) vs. non-users	–	–	1.34	[1.30, 1.37]	–	–	2.61	[2.45, 2.77]
	Age^a^	1.85	[1.84, 1.87]	1.91	[1.89, 1.93]	1.79	[1.76, 1.83]	1.80	[1.75, 1.84]
	Age-squared^a^	0.80	[0.80, 0.81]	0.80	[0.80, 0.80]	0.88	[0.87, 0.89]	0.88	[0.87, 0.89]
	Age-cohort								
	<1975	0.71	[0.69, 0.73]	0.67	[0.65, 0.69]	0.43	[0.40, 0.47]	0.44	[0.41, 0.48]
	1975–1979	0.71	[0.69, 0.73]	0.69	[0.67, 0.71]	0.60	[0.56, 0.65]	0.61	[0.56, 0.65]
	1980–1984	Ref		Ref		Ref		Ref	
	1985+	1.87	[1.82, 1.92]	1.92	[1.86, 1.97]	2.53	[2.38, 2.70]	2.59	[2.43, 2.78]

See Appendix D (Supplementary material) for rate within years.

a Age: per five year change.

b Model 1 calculates the rate ratio, comparing the historical offences of opiate users with non-users, controlling for all other variables in table.

c Model 2 includes linked data on age of opiate initiation, to model the effect of opiate initiation on the offending Rate Ratio. This allows the comparison of opiate users and non-users both prior to and post- opiate initiation.

#### 3.3.2 Model 2: change in offending trajectory accounting for initiation of drug use

The pre-initiation offending rate for male opiate-positive cases was double the rate for test-negative controls (RR = 2.00, 95% CI: 1.97–2.03), whilst the equivalent increased rate for females was 2.80 times (95% CI: 2.71–2.90). Initiation of opiate use increased the RR by 16% for males and 100% for females. Thus, the post-initiation rate was 2.32 times greater for cases than controls among males (95% CI: 2.29–2.35) and 5.61 times greater for females (95% CI: 5.47–5.75).

Both male and female cases had higher historical rates of non-serious and serious acquisitive offences prior to, and subsequent to, initiation of opiate use. For both serious and non-serious acquisitive offending categories and for both genders, initiation of opiate use increased the difference between cases and controls. Additionally, for both genders, there was a greater increase in the RR associated with initiation of opiate use for non-serious acquisitive crimes than serious crimes. In the case of violent offences, for females, the comparison between cases and controls was similar pre, and post, opiate-use initiation (RR: 2.51 and 2.61 respectively); the effect of opiate-use initiation in males was to reduce the RR (RR: 1.79 and 1.34).

We observed cohort effects; for example, controlling for age and drug-test status, later birth cohorts had higher rates of overall historical offending than earlier birth cohorts. However, this did not hold for the sub-categories of non-serious acquisitive crime, where each birth cohort had a similar rate of offending, or for serious acquisitive crime where, for men, earlier birth cohorts had a higher rate of offending.

A sensitivity analysis which separated the opiate-positive group into those that tested positive for opiates only and those that tested positive for opiates and cocaine, showed that the effect of opiate initiation was similar for both (see Appendix C in the Supplementary material).

## 4 Discussion

### 4.1 Summary of main findings

Those testing positive for opiates had substantially higher rates of prior sanctioned offending over their life-course than those testing negative for opiates and cocaine. This finding held for both males and females, whilst controlling for age and birth cohort. Findings support our four *a priori* hypotheses regarding offending prior to, and post, opiate-use initiation: 1) opiate–positives had higher rates of offending than test-negative controls prior to their opiate-use onset; 2) initiation of opiate use exacerbates existing levels of offending compared to controls; 3) initiation of opiate use was associated with a larger increase in the rate ratio (RR) for female than male users; 4) the effect of opiate-use initiation on historical offending differs by crime type as well as by gender.

Of particular interest is the RR reduction in violent offending associated with opiate use initiation observed in male users; while for female users, the RR was relatively unchanged. Opiate-use initiation was associated with greater elevation in non-serious (e.g., shop-lifting) than serious (e.g., burglary) acquisitive crime for both male and female users.

Our previous work demonstrated the association between opiate use and recent offending, whilst highlighting that the strength of the association varies by gender and offence type (Pierce et al., 2015). The present study expands on this analysis to investigate the longitudinal relationship between opiate-use initiation and crime. The majority of research carried out to examine the association between opiate use and crime has used a single cohort, pre/post design (Hayhurst et al., 2017), rather than a separate control group. Our use of offending records over the life-course, together with a suitable control group of non-using offenders, whilst also controlling for age and birth cohort, are all important design strengths. Additionally, we use a large sample size (n = 18,965 cases; n = 78,838 controls) to supply the necessary statistical power needed to detect differences differentiated by gender and sub-category of offending.

### 4.2 Limitations

The current study has some weaknesses. First, the use of a retrospective design limits the inferences that can be made – for instance, we cannot assess the influence that prior offending has on the likelihood of future opiate use. We are unable to hypothesise the extent to which offending prior to opiate-use initiation is associated with use of other substances, such as cannabis or alcohol, which may precede opiate use initiation (Lessem et al., 2006, Lynskey, 2003). Also, the opiate-using cohort may not be representative of opiate users in general. The cohort is sampled from individuals who received a drug test on arrest and were subsequently sanctioned; therefore, it is of greater relevance to opiate-using offenders.

The measures used are imperfect. Drug-using offenders may be more likely than non-users to be apprehended (Bond and Sheridan, 2007, Stevens, 2008) due, for example, to intoxication leading to easier identification. This may account for some of the differences detected in the current analysis, and, potentially, for differences in the period prior to initiation of opiate use, during which the likelihood of arrest may be affected by misuse of other substances, but this explanation is unlikely to account for the strength of the association observed here. Our work corresponds with previous research highlighting high levels of offending in opiate users prior to opiate-use onset (Shaffer et al., 1987); suggestive of common factors underlying both behaviours. Additionally, misclassification of non-cases was evident: 7% of negative testers were linked to an NDTMS record confirming drug-user status. Cases were identified via a saliva test which, despite having high sensitivity and specificity (Kacinko et al., 2004), only detects opiates used up to 24 h prior to testing(Verstraete, 2004) and so may not have identified less-problematic users. Any such misclassification would mean that the opiate-user and non-user group identified in this study are more similar than they would be under any ‘gold-standard’ testing procedure, meaning that the results presented are likely to be overly conservative, therefore not disputing our conclusions.

There was missing information on age of initiation for 33% of opiate positive testers; the majority because they did not have a treatment record over the data collection period. Secondary analysis of those with missing data (see Appendix A in the Supplementary material) showed that those who were not linked to NDTMS were less likely to test positive for both opiates and cocaine and were more likely to be male. Inspection of the graphs of offending rate by age group shows that those with missing linkage to NDTMS records had lower rates of offending over the life-course than those with complete information (see Appendix E in the Supplementary material). This could be because individuals who had not sought treatment were a shorter time into their using careers and not caught in a cycle of addiction and offending seen among those in this analysis. Therefore, the generalisability of these results might be affected by our focus on those individuals with a linked treatment record (75% of our cohort).

The findings of the present study are subject to unmeasured confounding. Information on important social factors, such as substance use or criminal behaviour among family members, was not available; neither was socio-economic status (Gauffin et al., 2013). However, even if suitable data were available, it may be difficult to establish the temporal ordering of change in socio-economic status and drug-use initiation.

### 4.3 Implications and findings in relation to other evidence

Our findings are directly relevant to Government drug policy as they are derived from individuals who have persisted in both their opiate use and offending. The findings confirm the relationship between opiate use and offending observed by others (Bennett et al., 2008, Bukten et al., 2011). We were also able to demonstrate that opiate-use onset is associated with crime escalation, independent of changes which occur with age. Therefore, initiation of opiate use appears to be a crucial driver of offending; measures to reduce offending should include drug-use prevention.

Others have highlighted that onset substance use in offenders impedes the process of “maturing” out of crime described by the age-crime curve (Hussong et al., 2004, Ouimet and Le Blanc, 1996, Schroeder et al., 2007). Greater escalation of offending, compared to controls, post-opiate initiation, was seen in female than male users. This confirms the findings of a recent review, which indicated lower offence rates pre-opiate use in females than males but a greater escalation of crime subsequent to opiate-use onset in females (Hayhurst et al., 2017).

The absence of a relationship between violent crime and onset-opiate use in this study is of significance. Our previous work found a strong association between women testing positive for opiate use and recent violent offending, although such offences were only recorded in 8% of women (Pierce et al., 2015). The current study indicates no apparent increase in violent offending by women associated with opiate initiation, and a relative reduction in violent crime for men. This finding tallies with previous research indicating no confirmed relationship between violent crime and onset-substance use (Parker and Auerhahn, 1998, White and Gorman, 2000).

The large impact of opiate-use initiation on non-serious acquisitive crime mirrors that of our previous work, which demonstrated a rate of shoplifting in opiate users that was between 3.5 (males) and 4.7 (females) times that of non-using offenders (Pierce et al., 2015). These findings could be explained by opiate users focussing on criminal activity that generates sufficient income to support current drug use and that is within the skill set of the individual user (James et al., 1979).

### 4.4 Further research

Previous research indicated greater increases in offending levels post-opiate use in individuals with onset of opiate use at an earlier age (Hayhurst et al., 2017). This corresponds with key offending theories in demonstrating that early antisocial or delinquent behaviour is associated with a more pronounced offending trajectory (Moffitt, 1993). It would be informative to examine this interaction further with the use of a control cohort. It would also be advantageous to analyse prospective, longitudinal cohorts so that information could be incorporated on those who desist in their offending and opiate use.

### 4.5 Conclusions

We have previously highlighted a surprising lack of high-quality research with which to delineate the nature of the relationship between drug use, in general, and opiate use, in particular, and crime. This is one of a handful of studies to employ a control group to account for the well-known relationship between age, drug use and crime. Findings indicate a more complex drugs-crime relationship than that espoused by current drug policy (Home Office, 2010) with already higher than expected levels of offending in those who go on to use drugs, such as opiates, problematically and whose offending behaviour then escalates. Having a more nuanced understanding of the nature of the drugs-crime relationship is crucial to the development of policy responses underpinning decisions about how best to intervene to interrupt the pathway from onset crime to onset substance use (Hayhurst et al., 2017). Findings suggest that complex interventions that target young, particularly female, offenders are required. Indeed, our findings align with the conclusions of others who have suggested that it is quite viable to identify future problematic substance users by patterns of early-life delinquent and offending behaviour, allowing for targeted intervention (Macleod et al., 2013).

## Funding

This research was funded as part of the Insights study by the UK Medical Research Council (MR/J013560/1). The MRC had no further role in study design; in the collection, analysis and interpretation of data; in the writing of the report; or in the decision to submit the paper for publication. The Home Office have been provided with a pre-submission version of this manuscript but have not exerted any editorial control over, or commented on, its content. Sheila Bird is funded by Medical Research Council programme number MC_U105260794.

## Contributors

**Millar**, **Pierce** and **Hayhurst** conceived of the study. **Pierce** with input from **Bird** wrote the analysis plan. **Pierce** analysed the data and wrote a first draft of the manuscript. **Millar**, **Bird** and **Dunn** supervised data analysis. All interpreted the data, edited, and approved of the manuscript.

## Conflicts of interest

**Millar** has received research funding from the UK National Treatment Agency for Substance Misuse and the Home Office. He has been a member of the organising committee for conferences supported by unrestricted educational grants from Reckitt Benckiser, Lundbeck, Martindale Pharma, and Britannia Pharmaceuticals Ltd, for which he received no personal remuneration. He is a member of the Advisory Council on the Misuse of Drugs. **Bird** holds GSK shares. She is formerly an MRC programme leader and has been elected to Honorary Professorship at Edinburgh University. She chaired Home Office’s Surveys, Design and Statistics Subcommittee (SDSSC) when SDSSC published its report on 21st Century Drugs and Statistical Science. She has previously served as UK representative on the Scientific Committee for European Monitoring Centre for Drugs and Drug Addiction. She is co-principal investigator for MRC-funded, prison-based N-ALIVE pilot Trial. **Seddon** has received research funding from the UK National Treatment Agency for Substance Misuse and the Home Office. **Hayhurst** has received grant research funding from Change, Grow, Live (CGL), a 3rd-sector provider of substance misuse services.
