# Supplementary material for: Insights into the link between drug use and criminality: Lifetime offending of criminally-active opiate users
Source: Drug Alcohol Depend. 2017 Oct 1;179:309–16. doi: 10.1016/j.drugalcdep.2017.07.024 (PMC5608072; doi:10.1016/j.drugalcdep.2017.07.024)

**Supplementary Material for the Article:**

Insights into the link between drug use and criminality:

Lifetime offending of criminally-active opiate users*

Matthias Pierce^a^, *matthias.pierce@manchester.ac.uk*

Karen Hayhurst^a^, *karen.hayhurst@manchester.ac.uk*

Sheila M Bird^b^, *sheila.bird@mrc-bsu.cam.ac.uk*

Matthew Hickman^c^, *matthew.hickman@bristol.ac.uk*

Toby Seddon^d^, *toby.seddon@manchester.ac.uk*

Graham Dunn^e^, *graham.dunn@manchester.ac.uk*

Tim Millar^a^, *tim.millar@manchester.ac.uk*

^a^ Centre for Mental Health and Safety, University of Manchester, 4th Floor, Ellen Wilkinson Building, Oxford Road, UK, M13 9PL

^b^ MRC Biostatistics Unit, Institute of Public Health, University Forvie Site, Robinson Way, Cambridge. UK, CB2 0SR

^c^ School of Social and Community Medicine, University of Bristol, Canynge Hall, 39 Whatley Road, Bristol, UK, BS8 2PS

^d^ School of Law, University of Manchester, 4.46A Williamson Building, Oxford Road, UK, M13 9PL

^e^ Centre for Biostatistics, University of Manchester, Jean McFarlane Building (First Floor), Oxford Road, UK, M13 9PL

**Correspondence:**

Matthias Pierce

Centre for Mental Health and Safety

University of Manchester

4th Floor, Ellen Wilkinson Building

Oxford Road, UK, M13 9PL

matthias.pierce@manchester.ac.uk

**This material supplements, but does not replace, the peer-reviewed paper in**

***Drug and Alcohol Dependence*.**

**Appendix A: Missing data description**

|  | Not missing (N=12,727) | Missing NDTMS data (N=1,708) | Missing NDTMS link (N=4,530) |
| --- | --- | --- | --- |
| Index test result |  |  |  |
| Opiate positive, cocaine negative | 4,780 (38) | 642 (38) | 1,837 (41) |
| Opiate positive, cocaine positive | 7,947 (62) | 1,066 (62) | 2,693 (59) |
| Gender |  |  |  |
| Male | 9,404 (73) | 1,300 (76) | 3,647 (81) |
| Female | 3,323 (26) | 408 (24) | 883 (19) |
| Median age at test [IQR] | 30.1 [26.2-34.5] | 31.4 [27.3-35.2] | 28.4 [23.6-33.9] |
| Median number crimes | 29 [12-54] | 28 [11-51] | 11 [2-34] |
| Median age at first recorded offence [IQR] | 16.7 [14.6-19.3] | 17.1 [14.7-19.5] | 17.1 [14.7-20.1] |

**Appendix B Offence classification**

| Offence group | Details of offences |
| --- | --- |
| **Serious acquisitive offences** |  |
| Burglary | Burglary in a dwelling; Aggravated burglary in a dwelling; Burglary other than in a dwelling; Aggravated burglary in a building other than a dwelling; Going equipped for stealing, etc, |
| Robbery | Robbery and assaults with intent to rob; |
| Theft of a vehicle | Aggravated vehicle taking; Stealing and unauthorised taking of motor vehicle |
| Stealing from vehicles: | From motor vehicles; From other vehicles |
| **Non-serious acquisitive offences** |  |
| Theft from shop | Stealing from shops and stalls |
| Other theft and handling stolen goods | Money laundering offences (not drugs); Stealing by an employee; Abstracting electricity; Stealing pedal cycles; Stealing from automatic machines and meters; Other theft or unauthorised taking; Handling stolen goods |
| Fraud and forgery | Frauds by company directors, etc; False accounting; Other fraud Bankruptcy and insolvency; Forgery, etc. of prescription; Other forgery, etc; Blackmail; |
| Prostitution |  |
| Theft from person | Stealing from the person of another; |
| Drug offences – supply | Including possession with intent |
| **Violence offences** | Murder; Attempted murder; Threats, conspiracy or incitement to murder; Manslaughter, etc; Wounding and other act endangering life; Endangering railway passenger: Endangering life at sea; Cruelty to or neglect of children; Abandoning children under two years; Child abduction; Procuring illegal abortion; Concealment of birth; common assault |

**Appendix C. Effect of opiate initiation for opiate-only users and opiate and cocaine users**

|  |  | Male | | Female | |
| --- | --- | --- | --- | --- | --- |
| Offence category | Variable | RR | 95% CI | RR | 95% CI |
| All crimes | Opiate-only users | 1.12 | [1.10, 1.14] | 2.15 | [2.05, 2.25] |
|  | Opiate and cocaine users | 1.18 | [1.17, 1.20] | 1.94 | [1.88, 1.99] |
|  |  |  |  |  |  |
| Non-serious acquisitive | Opiate-only users vs. non-users | 1.66 | [1.61, 1.71] | 2.46 | [2.32, 2.60] |
|  | Opiate and cocaine users vs. non-users | 1.76 | [1.72, 1.80] | 2.07 | [2.00, 2.15] |
|  |  |  |  |  |  |
| Serious acquisitive | Opiate-only users vs. non-users | 1.17 | [1.13, 1.20] | 1.73 | [1.50, 1.99] |
|  | Opiate and cocaine users vs. non-users | 1.30 | [1.27, 1.34] | 1.78 | [1.61, 1.96] |
|  |  |  |  |  |  |
|  |  |  |  |  |  |
| Violent offences | Opiate-only users vs. non-users | 0.73 | [0.70, 0.78] | 1.07 | [0.94, 1.22] |
|  | Opiate and cocaine users vs. non-users | 0.76 | [0.72, 0.79] | 1.03 | [0.93, 1.13] |
|  |  |  |  |  |  |

All analyses adjusted for age, age-squared and age cohort (<1975, 74-79, 80-84, 85+)

**Appendix D: offences grouped per year of age males**

|  | All crimes | | | | Violent crimes | | | | Serious acquisitive | | | | Serious non-acquisitive | | | |
| --- | --- | --- | --- | --- | --- | --- | --- | --- | --- | --- | --- | --- | --- | --- | --- | --- |
|  | Opiate users | | Non users | | Opiate users | | Non users | | Opiate users | | Non users | | Opiate users | | Non users | |
| Age | N | Rate [95% CI] | N | Rate [95% CI] | N | Rate [95% CI] | N | Rate [95% CI] | N | Rate [95% CI] |  |  |  |  |  |  |
| 10- | 744 | 0.05 [0.05, 0.06] | 2211 | 0.04 [0.04, 0.04] | 19 | 0.00 [0.00, 0.00] | 126 | 0.00 [0.00, 0.00] | 249 | 0.02 [0.02, 0.02] | 591 | 0.01 [0.01, 0.01] | 314 | 0.02 [0.02, 0.02] | 781 | 0.01 [0.01, 0.01] |
| 11- | 1698 | 0.12 [0.11, 0.12] | 5047 | 0.08 [0.08, 0.09] | 56 | 0.00 [0.00, 0.01] | 404 | 0.01 [0.01, 0.01] | 630 | 0.04 [0.04, 0.05] | 1310 | 0.02 [0.02, 0.02] | 663 | 0.05 [0.04, 0.05] | 1756 | 0.03 [0.03, 0.03] |
| 12- | 3893 | 0.27 [0.26, 0.28] | 10193 | 0.17 [0.17, 0.17] | 183 | 0.01 [0.01, 0.01] | 906 | 0.02 [0.01, 0.02] | 1417 | 0.10 [0.09, 0.10] | 2813 | 0.05 [0.05, 0.05] | 1480 | 0.10 [0.10, 0.11] | 3158 | 0.05 [0.05, 0.05] |
| 13- | 7389 | 0.51 [0.50, 0.53] | 19366 | 0.32 [0.32, 0.33] | 393 | 0.03 [0.02, 0.03] | 1785 | 0.03 [0.03, 0.03] | 2868 | 0.20 [0.19, 0.21] | 5847 | 0.10 [0.09, 0.10] | 2205 | 0.15 [0.15, 0.16] | 5041 | 0.08 [0.08, 0.09] |
| 14- | 14104 | 0.98 [0.97, 1.00] | 35525 | 0.59 [0.59, 0.60] | 783 | 0.05 [0.05, 0.06] | 3117 | 0.05 [0.05, 0.05] | 5350 | 0.37 [0.36, 0.38] | 11169 | 0.19 [0.18, 0.19] | 3462 | 0.24 [0.23, 0.25] | 7602 | 0.13 [0.12, 0.13] |
| 15- | 21680 | 1.51 [1.49, 1.53] | 53894 | 0.90 [0.89, 0.91] | 1175 | 0.08 [0.08, 0.09] | 4726 | 0.08 [0.08, 0.08] | 8037 | 0.56 [0.55, 0.57] | 15618 | 0.26 [0.26, 0.26] | 4752 | 0.33 [0.32, 0.34] | 9789 | 0.16 [0.16, 0.17] |
| 16- | 29362 | 2.05 [2.02, 2.07] | 72671 | 1.21 [1.20, 1.22] | 1594 | 0.11 [0.11, 0.12] | 5460 | 0.09 [0.09, 0.09] | 9968 | 0.69 [0.68, 0.71] | 19155 | 0.32 [0.31, 0.32] | 6069 | 0.42 [0.41, 0.43] | 11616 | 0.19 [0.19, 0.20] |
| 17- | 37046 | 2.58 [2.56, 2.61] | 91317 | 1.52 [1.51, 1.53] | 1861 | 0.13 [0.12, 0.14] | 6495 | 0.11 [0.11, 0.11] | 11293 | 0.79 [0.77, 0.80] | 20510 | 0.34 [0.34, 0.35] | 7843 | 0.55 [0.53, 0.56] | 14672 | 0.24 [0.24, 0.25] |
| 18- | 37216 | 2.62 [2.59, 2.64] | 82368 | 1.50 [1.49, 1.51] | 1896 | 0.13 [0.13, 0.14] | 5922 | 0.11 [0.11, 0.11] | 9462 | 0.67 [0.65, 0.68] | 15893 | 0.29 [0.28, 0.29] | 8594 | 0.60 [0.59, 0.62] | 13887 | 0.25 [0.25, 0.26] |
| 19- | 35874 | 2.57 [2.54, 2.59] | 67327 | 1.40 [1.39, 1.41] | 1731 | 0.12 [0.12, 0.13] | 4677 | 0.10 [0.09, 0.10] | 7853 | 0.56 [0.55, 0.57] | 11589 | 0.24 [0.24, 0.25] | 8753 | 0.63 [0.61, 0.64] | 12249 | 0.26 [0.25, 0.26] |
| 20- | 34535 | 2.52 [2.49, 2.55] | 56736 | 1.33 [1.32, 1.34] | 1643 | 0.12 [0.11, 0.13] | 3971 | 0.09 [0.09, 0.10] | 6801 | 0.50 [0.48, 0.51] | 8771 | 0.21 [0.20, 0.21] | 9210 | 0.67 [0.66, 0.69] | 11098 | 0.26 [0.25, 0.26] |
| 21- | 34037 | 2.54 [2.51, 2.57] | 49087 | 1.28 [1.27, 1.29] | 1629 | 0.12 [0.12, 0.13] | 3430 | 0.09 [0.09, 0.09] | 5973 | 0.45 [0.43, 0.46] | 6867 | 0.18 [0.17, 0.18] | 9489 | 0.71 [0.69, 0.72] | 10396 | 0.27 [0.27, 0.28] |
| 22- | 31409 | 2.41 [2.39, 2.44] | 43158 | 1.25 [1.24, 1.26] | 1521 | 0.12 [0.11, 0.12] | 2950 | 0.09 [0.08, 0.09] | 4967 | 0.38 [0.37, 0.39] | 5653 | 0.16 [0.16, 0.17] | 9496 | 0.73 [0.71, 0.74] | 9895 | 0.29 [0.28, 0.29] |
| 23- | 30275 | 2.42 [2.39, 2.44] | 36820 | 1.19 [1.18, 1.20] | 1318 | 0.11 [0.10, 0.11] | 2543 | 0.08 [0.08, 0.09] | 4510 | 0.36 [0.35, 0.37] | 4449 | 0.14 [0.14, 0.15] | 9676 | 0.77 [0.76, 0.79] | 8892 | 0.29 [0.28, 0.29] |
| 24- | 28074 | 2.35 [2.32, 2.38] | 32027 | 1.15 [1.14, 1.17] | 1293 | 0.11 [0.10, 0.11] | 2199 | 0.08 [0.08, 0.08] | 3920 | 0.33 [0.32, 0.34] | 3782 | 0.14 [0.13, 0.14] | 9012 | 0.75 [0.74, 0.77] | 7835 | 0.28 [0.28, 0.29] |
| 25- | 26507 | 2.35 [2.32, 2.38] | 28287 | 1.14 [1.12, 1.15] | 1177 | 0.10 [0.10, 0.11] | 1833 | 0.07 [0.07, 0.08] | 3448 | 0.31 [0.30, 0.32] | 3122 | 0.13 [0.12, 0.13] | 8646 | 0.77 [0.75, 0.78] | 7039 | 0.28 [0.28, 0.29] |
| 26- | 24701 | 2.35 [2.32, 2.38] | 24383 | 1.10 [1.08, 1.11] | 1032 | 0.10 [0.09, 0.10] | 1620 | 0.07 [0.07, 0.08] | 3172 | 0.30 [0.29, 0.31] | 2482 | 0.11 [0.11, 0.12] | 8281 | 0.79 [0.77, 0.81] | 6326 | 0.28 [0.28, 0.29] |
| 27- | 21874 | 2.27 [2.24, 2.30] | 21336 | 1.08 [1.06, 1.09] | 989 | 0.10 [0.10, 0.11] | 1572 | 0.08 [0.08, 0.08] | 2702 | 0.28 [0.27, 0.29] | 2223 | 0.11 [0.11, 0.12] | 7340 | 0.76 [0.75, 0.78] | 5477 | 0.28 [0.27, 0.28] |
| 28- | 19706 | 2.26 [2.23, 2.29] | 18409 | 1.05 [1.03, 1.06] | 816 | 0.09 [0.09, 0.10] | 1410 | 0.08 [0.08, 0.08] | 2328 | 0.27 [0.26, 0.28] | 1711 | 0.10 [0.09, 0.10] | 6936 | 0.80 [0.78, 0.82] | 4882 | 0.28 [0.27, 0.29] |
| 29- | 17761 | 2.27 [2.23, 2.30] | 16272 | 1.05 [1.04, 1.07] | 790 | 0.10 [0.09, 0.11] | 1178 | 0.08 [0.07, 0.08] | 1848 | 0.24 [0.23, 0.25] | 1391 | 0.09 [0.09, 0.09] | 6250 | 0.80 [0.78, 0.82] | 4574 | 0.30 [0.29, 0.30] |
| 30- | 15110 | 2.16 [2.13, 2.20] | 14488 | 1.07 [1.05, 1.08] | 737 | 0.11 [0.10, 0.11] | 1114 | 0.08 [0.08, 0.09] | 1615 | 0.23 [0.22, 0.24] | 1222 | 0.09 [0.08, 0.10] | 5190 | 0.74 [0.72, 0.76] | 3823 | 0.28 [0.27, 0.29] |
| 31- | 13043 | 2.13 [2.09, 2.16] | 12443 | 1.05 [1.04, 1.07] | 586 | 0.10 [0.09, 0.10] | 895 | 0.08 [0.07, 0.08] | 1330 | 0.22 [0.21, 0.23] | 966 | 0.08 [0.08, 0.09] | 4590 | 0.75 [0.73, 0.77] | 3390 | 0.29 [0.28, 0.30] |
| 32- | 11180 | 2.11 [2.08, 2.15] | 10693 | 1.06 [1.04, 1.08] | 521 | 0.10 [0.09, 0.11] | 800 | 0.08 [0.07, 0.08] | 1056 | 0.20 [0.19, 0.21] | 755 | 0.07 [0.07, 0.08] | 3937 | 0.74 [0.72, 0.77] | 2928 | 0.29 [0.28, 0.30] |
| 33- | 9292 | 2.06 [2.02, 2.10] | 8907 | 1.04 [1.02, 1.06] | 430 | 0.10 [0.09, 0.10] | 684 | 0.08 [0.07, 0.09] | 933 | 0.21 [0.19, 0.22] | 653 | 0.08 [0.07, 0.08] | 3213 | 0.71 [0.69, 0.74] | 2537 | 0.30 [0.28, 0.31] |
| 34- | 7678 | 2.06 [2.01, 2.10] | 7222 | 1.02 [1.00, 1.05] | 346 | 0.09 [0.08, 0.10] | 519 | 0.07 [0.07, 0.08] | 690 | 0.18 [0.17, 0.20] | 486 | 0.07 [0.06, 0.08] | 2711 | 0.73 [0.70, 0.75] | 2098 | 0.30 [0.28, 0.31] |
| 35- | 5717 | 1.95 [1.90, 2.00] | 6003 | 1.06 [1.04, 1.09] | 253 | 0.09 [0.08, 0.10] | 487 | 0.09 [0.08, 0.09] | 494 | 0.17 [0.15, 0.18] | 443 | 0.08 [0.07, 0.09] | 1972 | 0.67 [0.64, 0.70] | 1662 | 0.29 [0.28, 0.31] |
| 36- | 3862 | 1.76 [1.71, 1.82] | 4624 | 1.08 [1.05, 1.11] | 230 | 0.10 [0.09, 0.12] | 375 | 0.09 [0.08, 0.10] | 328 | 0.15 [0.13, 0.17] | 319 | 0.07 [0.07, 0.08] | 1349 | 0.62 [0.58, 0.65] | 1349 | 0.32 [0.30, 0.33] |
| 37- | 2560 | 1.70 [1.63, 1.76] | 3137 | 1.05 [1.01, 1.09] | 146 | 0.10 [0.08, 0.11] | 253 | 0.08 [0.07, 0.10] | 233 | 0.15 [0.14, 0.18] | 176 | 0.06 [0.05, 0.07] | 975 | 0.65 [0.61, 0.69] | 1005 | 0.34 [0.32, 0.36] |
| 38- | 1356 | 1.57 [1.49, 1.66] | 2071 | 1.19 [1.14, 1.24] | 77 | 0.09 [0.07, 0.11] | 213 | 0.12 [0.11, 0.14] | 121 | 0.14 [0.12, 0.17] | 121 | 0.07 [0.06, 0.08] | 457 | 0.53 [0.48, 0.58] | 705 | 0.41 [0.38, 0.44] |
| 39- | 470 | 1.64 [1.50, 1.80] | 997 | 1.80 [1.69, 1.92] | 22 | 0.08 [0.05, 0.12] | 66 | 0.12 [0.09, 0.15] | 58 | 0.20 [0.15, 0.26] | 90 | 0.16 [0.13, 0.20] | 166 | 0.58 [0.49, 0.67] | 321 | 0.58 [0.52, 0.65] |

**Appendix D: offences grouped per year of age females**

|  | All crimes | | | | Violent crimes | | | | Serious acquisitive | | | | Serious non-acquisitive | | | |
| --- | --- | --- | --- | --- | --- | --- | --- | --- | --- | --- | --- | --- | --- | --- | --- | --- |
|  | Opiate users | | Non users | | Opiate users | | Non users | | Opiate users | | Non users | | Opiate users | | Non users | |
| Age | N | Rate [95% CI] | N | Rate [95% CI] | N | Rate [95% CI] | N | Rate [95% CI] | N | Rate [95% CI] |  |  |  |  |  |  |
| 10- | 29 | 0.01 [0.00, 0.01] | 81 | 0.00 [0.00, 0.01] | 1 | 0.00 [0.00, 0.00] | 5 | 0.00 [0.00, 0.00] | 6 | 0.00 [0.00, 0.00] | 6 | 0.00 [0.00, 0.00] | 19 | 0.00 [0.00, 0.01] | 58 | 0.00 [0.00, 0.00] |
| 11- | 106 | 0.02 [0.02, 0.03] | 293 | 0.02 [0.01, 0.02] | 11 | 0.00 [0.00, 0.00] | 33 | 0.00 [0.00, 0.00] | 11 | 0.00 [0.00, 0.00] | 22 | 0.00 [0.00, 0.00] | 78 | 0.02 [0.01, 0.02] | 194 | 0.01 [0.01, 0.01] |
| 12- | 368 | 0.08 [0.07, 0.09] | 757 | 0.04 [0.04, 0.04] | 52 | 0.01 [0.01, 0.01] | 89 | 0.00 [0.00, 0.01] | 40 | 0.01 [0.01, 0.01] | 92 | 0.00 [0.00, 0.01] | 206 | 0.04 [0.04, 0.05] | 458 | 0.02 [0.02, 0.03] |
| 13- | 899 | 0.19 [0.18, 0.21] | 1622 | 0.09 [0.08, 0.09] | 138 | 0.03 [0.03, 0.04] | 294 | 0.02 [0.01, 0.02] | 142 | 0.03 [0.03, 0.04] | 160 | 0.01 [0.01, 0.01] | 404 | 0.09 [0.08, 0.10] | 763 | 0.04 [0.04, 0.04] |
| 14- | 1783 | 0.39 [0.37, 0.40] | 3394 | 0.18 [0.17, 0.19] | 278 | 0.06 [0.05, 0.07] | 629 | 0.03 [0.03, 0.04] | 244 | 0.05 [0.05, 0.06] | 392 | 0.02 [0.02, 0.02] | 772 | 0.17 [0.16, 0.18] | 1360 | 0.07 [0.07, 0.08] |
| 15- | 2602 | 0.56 [0.54, 0.59] | 4911 | 0.26 [0.25, 0.27] | 381 | 0.08 [0.07, 0.09] | 887 | 0.05 [0.04, 0.05] | 307 | 0.07 [0.06, 0.07] | 485 | 0.03 [0.02, 0.03] | 1128 | 0.24 [0.23, 0.26] | 1872 | 0.10 [0.09, 0.10] |
| 16- | 3869 | 0.84 [0.81, 0.87] | 5781 | 0.31 [0.30, 0.31] | 388 | 0.08 [0.08, 0.09] | 821 | 0.04 [0.04, 0.05] | 359 | 0.08 [0.07, 0.09] | 450 | 0.02 [0.02, 0.03] | 1966 | 0.43 [0.41, 0.45] | 2317 | 0.12 [0.12, 0.13] |
| 17- | 6038 | 1.31 [1.28, 1.34] | 7542 | 0.40 [0.39, 0.41] | 332 | 0.07 [0.06, 0.08] | 797 | 0.04 [0.04, 0.05] | 345 | 0.07 [0.07, 0.08] | 527 | 0.03 [0.03, 0.03] | 3517 | 0.76 [0.74, 0.79] | 3386 | 0.18 [0.17, 0.19] |
| 18- | 7553 | 1.66 [1.62, 1.70] | 7362 | 0.41 [0.41, 0.42] | 316 | 0.07 [0.06, 0.08] | 672 | 0.04 [0.04, 0.04] | 374 | 0.08 [0.07, 0.09] | 327 | 0.02 [0.02, 0.02] | 4319 | 0.95 [0.92, 0.98] | 3568 | 0.20 [0.19, 0.21] |
| 19- | 7998 | 1.80 [1.76, 1.84] | 7153 | 0.45 [0.44, 0.46] | 286 | 0.06 [0.06, 0.07] | 556 | 0.03 [0.03, 0.04] | 326 | 0.07 [0.07, 0.08] | 294 | 0.02 [0.02, 0.02] | 4588 | 1.03 [1.00, 1.06] | 3667 | 0.23 [0.22, 0.24] |
| 20- | 8559 | 1.99 [1.94, 2.03] | 6569 | 0.46 [0.45, 0.47] | 277 | 0.06 [0.06, 0.07] | 389 | 0.03 [0.02, 0.03] | 278 | 0.06 [0.06, 0.07] | 210 | 0.01 [0.01, 0.02] | 4899 | 1.14 [1.10, 1.17] | 3712 | 0.26 [0.25, 0.27] |
| 21- | 8644 | 2.09 [2.05, 2.13] | 6440 | 0.50 [0.48, 0.51] | 265 | 0.06 [0.06, 0.07] | 370 | 0.03 [0.03, 0.03] | 257 | 0.06 [0.05, 0.07] | 163 | 0.01 [0.01, 0.01] | 4995 | 1.21 [1.17, 1.24] | 3556 | 0.27 [0.26, 0.28] |
| 22- | 8768 | 2.22 [2.18, 2.27] | 5814 | 0.49 [0.48, 0.50] | 308 | 0.08 [0.07, 0.09] | 321 | 0.03 [0.02, 0.03] | 243 | 0.06 [0.05, 0.07] | 156 | 0.01 [0.01, 0.02] | 5001 | 1.27 [1.23, 1.30] | 3237 | 0.27 [0.26, 0.28] |
| 23- | 8255 | 2.22 [2.17, 2.27] | 5409 | 0.51 [0.49, 0.52] | 232 | 0.06 [0.05, 0.07] | 290 | 0.03 [0.02, 0.03] | 240 | 0.06 [0.06, 0.07] | 141 | 0.01 [0.01, 0.02] | 4676 | 1.26 [1.22, 1.29] | 3049 | 0.28 [0.27, 0.29] |
| 24- | 7454 | 2.16 [2.12, 2.21] | 4971 | 0.51 [0.50, 0.53] | 199 | 0.06 [0.05, 0.07] | 264 | 0.03 [0.02, 0.03] | 222 | 0.06 [0.06, 0.07] | 119 | 0.01 [0.01, 0.01] | 4253 | 1.23 [1.20, 1.27] | 2709 | 0.28 [0.27, 0.29] |
| 25- | 7079 | 2.23 [2.17, 2.28] | 4590 | 0.52 [0.51, 0.54] | 209 | 0.07 [0.06, 0.08] | 198 | 0.02 [0.02, 0.03] | 194 | 0.06 [0.05, 0.07] | 71 | 0.01 [0.01, 0.01] | 3888 | 1.22 [1.18, 1.26] | 2547 | 0.29 [0.28, 0.30] |
| 26- | 6540 | 2.25 [2.20, 2.31] | 4391 | 0.55 [0.54, 0.57] | 178 | 0.06 [0.05, 0.07] | 210 | 0.03 [0.02, 0.03] | 177 | 0.06 [0.05, 0.07] | 88 | 0.01 [0.01, 0.01] | 3654 | 1.26 [1.22, 1.30] | 2552 | 0.32 [0.31, 0.34] |
| 27- | 6006 | 2.28 [2.22, 2.34] | 3519 | 0.50 [0.48, 0.51] | 156 | 0.06 [0.05, 0.07] | 169 | 0.02 [0.02, 0.03] | 147 | 0.06 [0.05, 0.07] | 87 | 0.01 [0.01, 0.02] | 3225 | 1.22 [1.18, 1.27] | 1999 | 0.28 [0.27, 0.30] |
| 28- | 5304 | 2.24 [2.18, 2.30] | 3216 | 0.51 [0.49, 0.53] | 137 | 0.06 [0.05, 0.07] | 149 | 0.02 [0.02, 0.03] | 136 | 0.06 [0.05, 0.07] | 68 | 0.01 [0.01, 0.01] | 2921 | 1.23 [1.19, 1.28] | 1778 | 0.28 [0.27, 0.30] |
| 29- | 4483 | 2.16 [2.10, 2.22] | 3079 | 0.55 [0.53, 0.57] | 133 | 0.06 [0.05, 0.08] | 152 | 0.03 [0.02, 0.03] | 101 | 0.05 [0.04, 0.06] | 47 | 0.01 [0.01, 0.01] | 2476 | 1.19 [1.15, 1.24] | 1638 | 0.29 [0.28, 0.31] |
| 30- | 3946 | 2.16 [2.09, 2.23] | 2494 | 0.51 [0.49, 0.53] | 126 | 0.07 [0.06, 0.08] | 131 | 0.03 [0.02, 0.03] | 66 | 0.04 [0.03, 0.05] | 50 | 0.01 [0.01, 0.01] | 2114 | 1.16 [1.11, 1.21] | 1314 | 0.27 [0.25, 0.28] |
| 31- | 3297 | 2.06 [1.99, 2.13] | 2218 | 0.51 [0.49, 0.53] | 109 | 0.07 [0.06, 0.08] | 149 | 0.03 [0.03, 0.04] | 57 | 0.04 [0.03, 0.05] | 45 | 0.01 [0.01, 0.01] | 1795 | 1.12 [1.07, 1.17] | 1153 | 0.27 [0.25, 0.28] |
| 32- | 2870 | 2.09 [2.01, 2.17] | 2046 | 0.54 [0.52, 0.57] | 92 | 0.07 [0.05, 0.08] | 154 | 0.04 [0.03, 0.05] | 64 | 0.05 [0.04, 0.06] | 45 | 0.01 [0.01, 0.02] | 1524 | 1.11 [1.05, 1.17] | 1008 | 0.27 [0.25, 0.28] |
| 33- | 2582 | 2.23 [2.15, 2.32] | 1812 | 0.56 [0.53, 0.59] | 70 | 0.06 [0.05, 0.08] | 121 | 0.04 [0.03, 0.04] | 60 | 0.05 [0.04, 0.07] | 45 | 0.01 [0.01, 0.02] | 1374 | 1.19 [1.13, 1.25] | 929 | 0.29 [0.27, 0.31] |
| 34- | 1865 | 2.02 [1.93, 2.12] | 1593 | 0.59 [0.56, 0.62] | 51 | 0.06 [0.04, 0.07] | 93 | 0.03 [0.03, 0.04] | 45 | 0.05 [0.04, 0.07] | 50 | 0.02 [0.01, 0.02] | 1005 | 1.09 [1.02, 1.16] | 832 | 0.31 [0.29, 0.33] |
| 35- | 1425 | 1.96 [1.86, 2.07] | 1263 | 0.59 [0.55, 0.62] | 46 | 0.06 [0.05, 0.08] | 82 | 0.04 [0.03, 0.05] | 35 | 0.05 [0.03, 0.07] | 19 | 0.01 [0.01, 0.01] | 757 | 1.04 [0.97, 1.12] | 651 | 0.30 [0.28, 0.33] |
| 36- | 938 | 1.75 [1.64, 1.86] | 980 | 0.60 [0.57, 0.64] | 30 | 0.06 [0.04, 0.08] | 76 | 0.05 [0.04, 0.06] | 11 | 0.02 [0.01, 0.04] | 15 | 0.01 [0.01, 0.02] | 511 | 0.95 [0.87, 1.04] | 503 | 0.31 [0.28, 0.34] |
| 37- | 614 | 1.71 [1.58, 1.85] | 590 | 0.52 [0.48, 0.56] | 26 | 0.07 [0.05, 0.11] | 44 | 0.04 [0.03, 0.05] | 14 | 0.04 [0.02, 0.07] | 9 | 0.01 [0.00, 0.02] | 325 | 0.90 [0.81, 1.01] | 307 | 0.27 [0.24, 0.30] |
| 38- | 359 | 1.81 [1.63, 2.01] | 429 | 0.64 [0.58, 0.70] | 10 | 0.05 [0.02, 0.09] | 30 | 0.04 [0.03, 0.06] | 8 | 0.04 [0.02, 0.08] | 7 | 0.01 [0.00, 0.02] | 182 | 0.92 [0.79, 1.06] | 263 | 0.39 [0.35, 0.44] |
| 39- | 103 | 1.72 [1.41, 2.09] | 206 | 0.93 [0.81, 1.07] | 3 | 0.05 [0.01, 0.15] | 17 | 0.08 [0.04, 0.12] | 0 | 0.00 [0.00, 0.06] | 4 | 0.02 [0.00, 0.05] | 65 | 1.09 [0.84, 1.39] | 138 | 0.62 [0.52, 0.74] |

**Figure Legends**

**Appendix Ea:** Offending rates by age-band for categories of offending and missingness, males

**Appendix Eb:** Offending rates by age-band for categories of offending and missingness, females

**Appendix Ea**


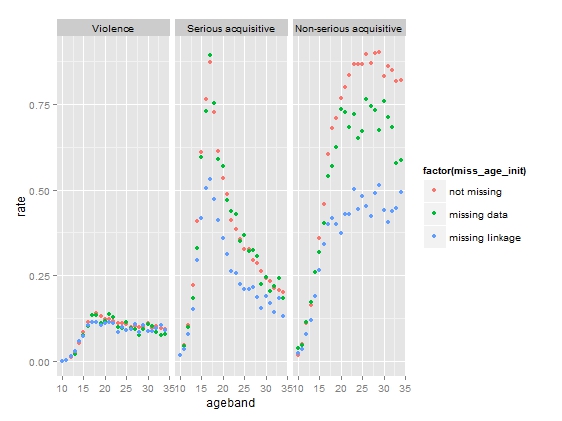


**Appendix Eb**


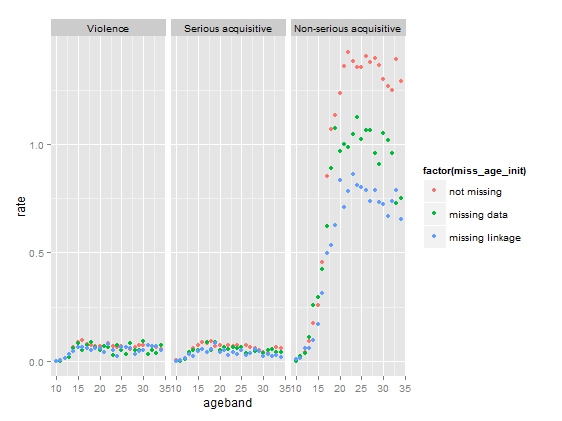

Supplement: Supplementary file 1 [file mmc1.docx]
